# Exploring the introduction of entrustment rating scales in an existing objective structured clinical examination

**DOI:** 10.1186/s12909-019-1736-2

**Published:** 2019-08-22

**Authors:** Ylva Holzhausen, Asja Maaz, Maren März, Victoria Sehy, Harm Peters

**Affiliations:** 1 Dieter Scheffner Center for Medical Education and Educational Research, Dean’s Office of Student Affairs, Charité – Universitätsmedizin Berlin, Free University of Berlin and Humboldt University of Berlin, Berlin, Germany; 2 Office of Student Affairs, Dean’s Office of Student Affairs, Charité – Universitätsmedizin Berlin, Free University of Berlin and Humboldt University of Berlin, Berlin, Germany

**Keywords:** Assessment of performance, Objective structured clinical examination, Entrustment scales, Entrustable professional activities

## Abstract

**Background:**

The concept of EPAs is increasingly applied to assess trainees’ workplace performance by means of entrustment ratings. OSCEs assess performance in a simulated setting, and it is unclear whether entrustment ratings can be integrated into these exams. This study explores the introduction of an entrustment rating scale into an existing OSCE.

**Methods:**

A 6-point entrustment scale was added to the standard ratings in an OSCE administered prior to students’ final clerkship year in an undergraduate medical programme. Standard OSCE ratings assess clinical and communication skills. Assessors (*n* = 54) rated students’ performance (*n* = 227) on a diverse set of clinical tasks and evaluated the addition of entrustment scales to OSCEs. Descriptive and inferential statistics were calculated for analyses.

**Results:**

Student performance varied across the stations, as reflected in both the standard OSCE ratings and the added entrustment ratings. Students received generally high standard OSCE ratings, whereas entrustment ratings were more widely distributed. All students passed the OSCE, and only a small proportion of students did not reach the expected pass threshold of 60% on the standard ratings in the single stations. The proportion of students who did not reach the expected entrustment level in the respective stations was noticeably higher. Both the clinical and communication skill ratings were related to the entrustment rating in most OSCE stations. A majority of the assessors positively evaluated the addition of entrustment ratings into the OSCE.

**Discussion:**

The findings provide an empirical basis to broaden our understanding of the potential use of entrustment ratings in existing OSCEs. They provide directions for future, more specific studies. The ratings might be used for formative feedback on students’ readiness for workplace practice.

## Background

The concept of entrustable professional activities (EPAs) has emerged as a new approach to the assessment of workplace performance [1–4]. Assessment through EPAs is centred on entrustment decisions by clinical supervisors that link trainees’ execution of professional activities with the level of supervision in their progress towards independent practice [5]. Objective structured clinical exams (OSCEs) assess the performance of medical trainees in a simulated setting. As OSCEs are widely used in medical education and the EPA concept is increasingly adopted as an overarching framework, it has been proposed to incorporate entrustment ratings, i.e., entrustment scales, into OSCE assessments [3, 6]. On theoretical grounds, there are pro and con arguments for doing so, while few studies actually report the results of entrustment ratings obtained in a simulated setting [7–9]. The present study aims to generate empirical insights into this matter by exploring the introduction of an entrustment scale into an existing undergraduate medical education OSCE.

Assessment of performance in competency-based medical education (CBME) – both in the workplace and in simulated settings – focuses on the assessment of trainees’ competencies [10]. A trainee needs to show the relevant and necessary skill, attitude or behaviour in interaction with a patient. The EPA concept was introduced to better operationalise the assessment of competencies in the workplace [5]. Instead of assessing relevant communication or clinical skills separately, a supervising physician evaluates a trainee by means of entrustment scales to determine how much supervision a trainee needs when he or she, for example, takes a medical history or performs a physical examination. Entrustment scales indicate a range of entrustment levels, indicating whether trainees can perform clinical tasks under close supervision, under moderate supervision or independently [11, 12]. Assessment with EPAs thus builds on the assessment of competencies but expands it by including the factors involved when clinical supervisors entrust a trainee to carry out certain tasks [13, 14].

While the EPA concept emphasises the clinical workplace, it also provides an overarching framework that allows a meaningful integration of workplace and non-workplace learning and assessments of medical trainees [12]. For instance, the results of knowledge tests and simulation-based performance assessments can be aligned with the scope or breadth of specific EPAs and thereby serve as a supporting information source that can feed into decisions on the workplace participation of trainees. One well-established and reliable mode to assess the performance of medical trainees in a simulated setting is the OSCE, which is widely used in undergraduate medical education [15]. In OSCEs, trainees perform certain clinical tasks, and their clinical and communication skills are assessed by means of either analytic checklists or holistic global rating scales [16, 17]. As the clinical tasks in OSCEs show great overlap with EPAs [1–4], it may be unsurprising that the inclusion of entrustment ratings is proposed for OSCEs [3, 6].

On theoretical grounds, one consideration that might favour the introduction of entrustment scales in OSCEs is that a rather minor modification of the existing assessment forms would allow OSCEs’ alignment with an overarching EPA framework. Entrustment scales could simply be added to existing marking schemes instead of developing and implementing completely new assessment forms. One may also argue that the addition of entrustment scales could potentially allow a more meaningful translation of OSCE results for workplace participation for both the trainee (“I am ready for indirect supervision”) and the clinical supervisor (“this trainee is ready for indirect supervision”).

On the contrary, there are considerations that would not support the introduction of entrustment scales in OSCEs. One is that performance on tasks in OSCEs does not involve any risk for a real patient, which is an important factor in entrustment decision-making. Furthermore, numerous other factors are controlled for the sake of standardisation in OSCEs, and these factors are relevant for entrustment decision-making in the workplace [13, 14]. Another potential problem is the brief contact between the trainee and the clinical assessor and the lack of a direct interaction. This limited interaction is sufficient to evaluate trainees’ competencies, but it may not be sufficient to evaluate their trustworthiness.

To date, little empirical research is available on the use of entrustment rating scales in the performance assessment of medical trainees in a simulated setting [7–9, 18, 19]. The aim of this study is to explore the introduction of an entrustment rating scale into a regular OSCE that students of an undergraduate medical programme must pass before they can enter the final-year clerkship. The main goal of this explorative study is to broaden our understanding and to prepare the groundwork for further studies. In particular, we aim to address the following three questions: 1) How does the distribution of standard OSCE ratings, i.e., on clinical and communication skills, including no-pass results, compare to ratings on entrustment scales? 2) How do standard OSCE ratings relate to entrustment ratings? 3) What do assessors think about introducing entrustment ratings into OSCEs?

## Methods

### Medical school setting

The integrated, competency-based undergraduate medical programme encompasses 6 years at the Charité - Universitätsmedizin Berlin (Charité), Germany. Performance-based exams are administered at the end of the 1st, 2nd and 5th years. Our study was incorporated into the obligatory and summative OSCE examination in the 5th year. Passing this OSCE is a requirement to enter the final clerkship year. The study protocol received approval from the assessment committee of the study programme as well as from the Charité data protection office and ethics board (No. EA4/136/18).

The OSCE consists of six stations in which students must perform a diverse set of clinical tasks. The content of each station is aligned with the learning objectives of the preceding semesters and includes interaction with standardised patients (SP) or with models. Students have ten minutes to complete each station, followed by a one-minute break. One rater per OSCE station assesses the performance of each student.

### Study protocol and participants

An entrustment rating scale was added to the standard marking scheme that assesses the performance of students. The OSCE assessors were asked to provide feedback on their perception and experience of incorporating an entrustment rating scale in the OSCE.

The results of all students taking part in the OSCE of July 2017 were collected. The OSCE took place on three consecutive days, with three parallel circuits. The circuits always had the same content and order of stations but different examiners and SPs. To add some unpredictability for the students, the task and content changed in two stations from one day to another. As a result, there were two versions of stations 1 and 5.

### Raters’ briefing

Before the start of the OSCE, the assessors received calibration training on the assessment of students’ performance and the application of the standard OSCE marking scheme. In addition, they were briefed about the purpose of the study and the application of the entrustment rating scale. They were informed that their entrustment rating was explorative and would not be reported to the students or be part of the students’ assessment results.

### Standard OSCE rating scales

The standard OSCE paper-based marking scheme consists of two components: one rating of clinical skills and one rating of communication skills. Clinical skills are rated by checklist items on a three-point scale (completely fulfilled = 1, partly fulfilled = 0.6, not fulfilled = 0). Communication skills are rated by an established global rating scale [17, 20]. The scale consists of four items: empathy (response to a patient’s feelings and needs), structure (internal consistency of a conversation), verbal expression and non-verbal expression. Students’ performance is rated for each item on a 5-point scale ranging from 1 = excellent to 5 = poor. Both the clinical and communication skill ratings are transformed into percent quotations and then combined into one composite OSCE rating per station, in which the clinical skill score has a weight of 70% and the communication skills score a weight of 30%. To calculate the overall OSCE result covering all stations, the results of all composite OSCE ratings per station are averaged. Students must reach at least 60% to pass the examination.

### Entrustment rating scale

The entrustment rating scale was provided on a separate paper sheet (see Table 1). Using a six-point scale, the assessors indicated how much supervision a student would need when performing the observed task in a clinical workplace. The entrustment levels were in line with recommendations in the literature [12, 21]. At the time of the OSCE, students are required to have spent a total of four months in short-term clerkships and been trained in bed-side teachings in the clinical activities that are part of OSCE. The minimal expected entrustment level to be reached was thus set to a level of 3 for the stations. The only exception was the OSCE station on necropsies, where a supervision level of 2 was expected.

**Table 1:** Entrustment rating scale. The letter L represents the word level

	The student is able to carry out the task …
L 1	in co-activity with the supervisor.
L 2	and the supervisor is present and steps in if needed.
L 3	autonomously, with supervision available within minutes and all findings being double-checked.
L 4	autonomously, with supervision available within minutes and key findings being double-checked.
L 5	autonomously, with supervision available but from a distance (e.g., by phone) and key findings being double-checked.
L 6	autonomously, with remote monitoring and key findings being reviewed.

### Assessors’ evaluation of incorporating entrustment scales into OSCEs

After the completion of all OSCE assessments, assessors completed a questionnaire with 5 items on the experienced usefulness of the added entrustment scale. Each item could be rated on a five-point scale (1 = I fully disagree – 5 = I fully agree).

### Statistical analysis

Analyses were carried out using SPSS 25 [22] and R 3.4.3 [23]. To answer research questions one and three, descriptive statistics were calculated for all OSCE scores on student performance and the assessor evaluation questionnaire. Violin plots were created to better compare the data distribution of standard OSCE scale ratings and entrustment ratings [24]. Linear mixed-effect models were used to estimate the differences in both the OSCE scales and the entrustment ratings between the six stations. The students were defined as the subjects, the stations were defined as both the repeated measure and the fixed factor, and the OSCE scales and the entrustment ratings were defined as the dependent variables.

To answer research question two, correlation and regression analyses were utilised for each OSCE station to estimate the relationship between the standard OSCE scale ratings and the entrustment scale rating. The standard OSCE scales were first correlated with the entrustment rating to determine whether a linear regression analysis was reasonable. The entrustment rating was entered as the dependent variable.

## Results

### Participants

In total, 227 students were assessed in the OSCE. Students were, on average, 26 years old (SD = 4), and 60% were female. One assessor of the first station did not complete the entrustment scales, which resulted in missing values for 25 students. In combination with seven randomly occurring missing values for the entrustment scale, this resulted in 2% missing values. In total, 54 physicians from various clinical disciplines at the Charité represented the assessors in the OSCE; 50% of them were female.

### Distribution of standard OSCE and entrustment ratings

Table 2 depicts a numeric overview of the OSCE stations’ titles and the rating results per station of the clinical and communication skills, the composite OSCE result and the entrustment rating.

**Table 2:** Descriptive statistics of standard OSCE ratings and entrustment ratings

	Station	Clinical skills rating	Communication skills rating	Composite OSCE result	Entrustment rating	*n* =
1.1	Humerus Fracture	73.1 (14.5)	87.9 (12.6)	77.5 (12.9)	4.0 (1.6)	71
1.2	Herniated Disc	81.8 (16.3)	78.0 (16.6)	80.6 (14.3)	3.8 (1.3)	156
2	Trigeminal Neuralgia	84.4 (11.7)	88.6 (12.6)	85.7 (9.7)	4.1 (1.4)	227
3	Depression	81.2 (11.5)	87.6 (12.4)	83.1 (9.9)	4.2 (1.1)	227
4	Paediatric Check-Up	86.1 (11.0)	86.6 (15.1)	86.3 (10.7)	3.7 (1.3)	227
5.1	Prostatic Hypertrophy	84.7 (9.0)	86.0 (14.0)	85.1 (8.2)	3.7 (1.3)	80
5.2	Falling	83.0 (12.1)	91.2 (9.1)	85.5 (9.5)	4.2 (.8)	147
6	Necropsy	92.0 (6.8)	/	92.0 (6.8)	3.3 (1.5)	227
Sum		84.4 (5.5)	86.7 (7.1)	85.3 (5.0)	3.9 (.6)	

Legend: Note: Mean percent scores (SD) are shown for the standard OSCE ratings (percent) and the mean score (SD) for the entrustment rating (scale 1–6)

The students reached a mean overall OSCE result of M = 83.5% (SD =5.0%). The mean entrustment rating was M = 3.9 (SD = 0.6) for all OSCE stations. Students’ performance varied between the OSCE stations, as reflected by differences in the composite OSCE ratings [F (1, 7) = 44.7, *p* < 0.001] and the entrustment ratings [F (1, 7) = 27.5, *p* < 0.001].

**Fig. 1:**
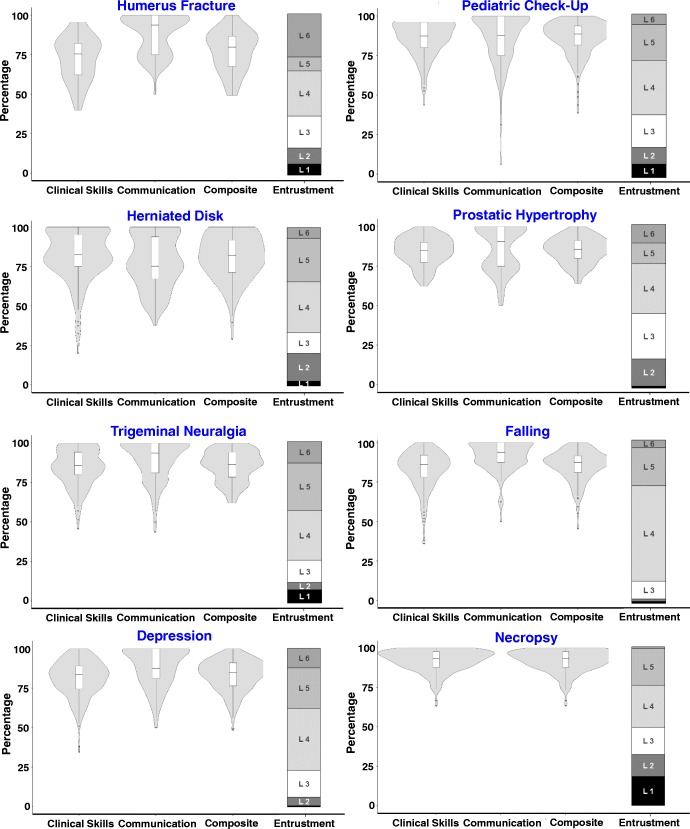
Vioplots (boxplots with kernel density plots; box plots indicate the median and 25 and 75% percentiles) of the standard OSCE ratings (clinical skills, communication skills and composite OSCE result) and bar plots of the distribution of entrustment scale ratings (for definitions of L1 to L6, see Table 1)

Figure 1 displays violin plots of the distribution of the standard OSCE ratings on clinical skills, communication skills and the composite result per OSCE station as well as the distribution of the entrustment ratings. The standard OSCE ratings show some variation between the stations and their topics. However, in general, the ratings on the clinical and communication skills and their composite tend to be located at the upper end of the scales. The entrustment ratings vary between the stations but cover the full scale and tend to be more evenly distributed.

**Fig. 2:**
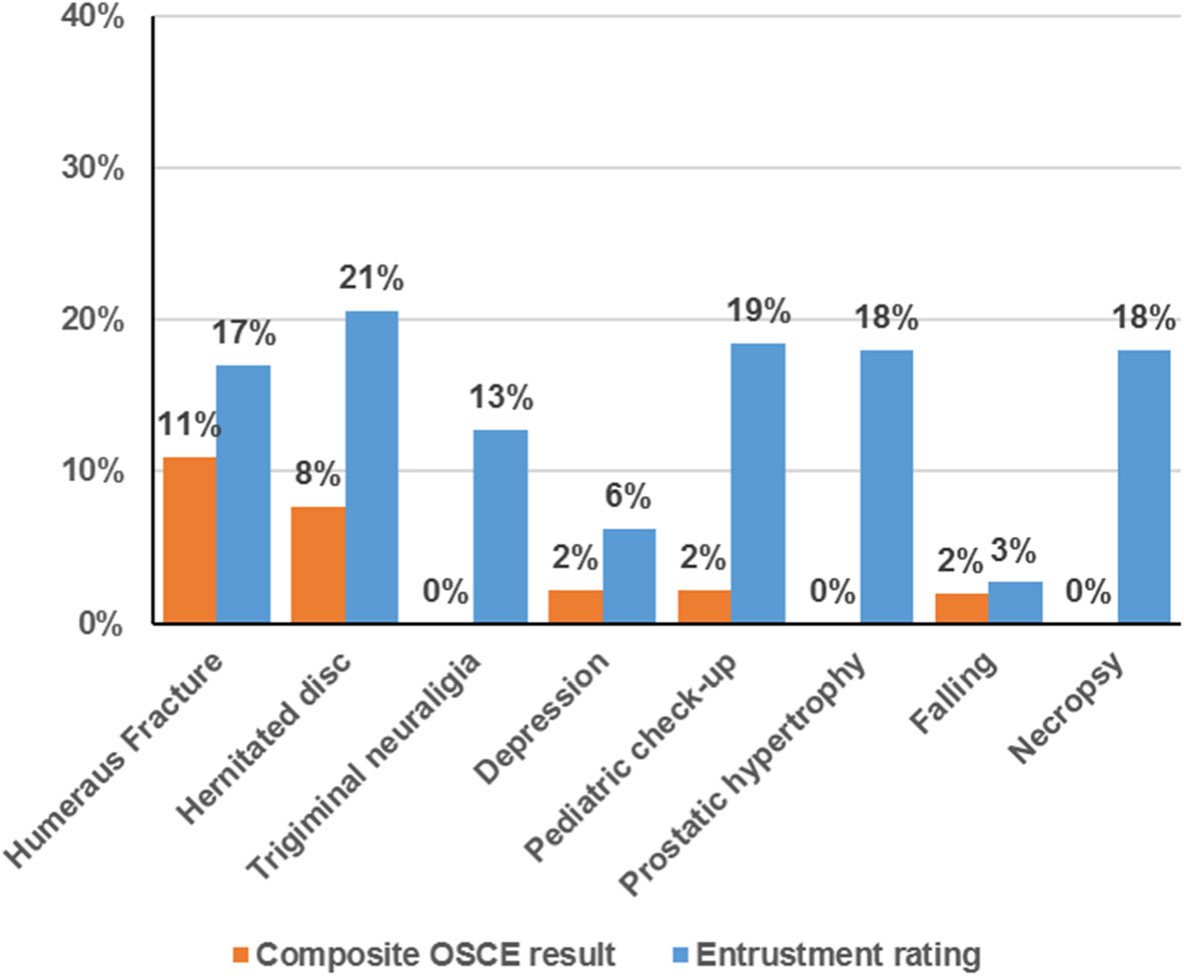
Percent of students who fail to reach the threshold level per station on the composite OSCE result (at least 60%) and the entrustment rating (at least level 3 in all stations but the necropsy station, where at least level 2 is required)

To further explore our data, we looked at the proportion of students under a minimum requirement threshold both for the full OSCE exam and for each OSCE station. The standard pass threshold for the OSCE at Charité is set to 60% for the overall OSCE result. All students managed to reach this threshold and hence pass the exam. Using the same 60% threshold for each station, there is some variability in the percentage of students who failed a station (Fig. 2). Analysis indicates that 12% (*n* = 27) of the students failed one station and 1% failed two stations (*n* = 3).

The expectation for the entrustment rating in the majority of the stations would be to carry out the respective task without direct supervision, which would correspond to our rating scale of 3 and higher. Only the necropsy station covered a more complex task, and the expected entrustment level was set to 2 for this station. The percentage of students failing to reach the expected entrustment level differs considerably per station, ranging between 3 and 21% (Fig. 2); 31% of the students did not reach the expected supervision level in at least one station, 15% in two stations and 6% in three stations or more.

### Relationship between standard OSCE and entrustment ratings

In the humerus fracture station, the clinical skill rating and communication skill rating do not correlate significantly with the entrustment rating. In the prostatic hypertrophy station, the clinical skill rating shows no significant correlation but the communication skill rating shows a significant negative correlation (r = − 0.362, *p* < 0.01) with the entrustment rating. No linear regression analyses are conducted for these stations.

Table 3 shows that the OSCE standard rating accounts for the variance in the entrustment rating in each station, whereas the degree varies. In the trigeminal neuralgia station, the standard OSCE scales account for 51% of the variance in the entrustment rating [R^2^ = 0.52; F [1222] = 119.25; *p* < 0.001), whereas the explained variance is 15% in the depression station [R^2^ = 0.15; F [1224] = 21.26; *p* < 0.001). In the necropsy station, only the clinical skill rating was applied, and the regression analysis shows the lowest amount of explained variance of the entrustment rating [R^2^ = 0.03; F [1221] = 8.41; *p* < 0.01).

**Table 3:** Regression analysis of the OSCE scales on the entrustment rating per station

Station		Model			Regression coefficients				
		R^2^	ΔR2	F		B	SE	β	t
1.1	Humerus Fracture								
1.2	Herniated Disc	.35	.34	34.11***	Checklist Score	.01	.01	.11	1.41
					Global Rating	.04	.01	.53	6.75***
2	Trigeminal neuralgia	.16	.15	21.26***	Checklist Score	.04	.01	.3	4.70***
					Global Rating	.02	.01	.21	3.32**
3	Depression	.52	.51	119.25***	Checklist Score	.03	.01	.35	7.06***
					Global Rating	.05	.00	.52	10.57***
4	Paediatric Check-Up	.44	.44	88.18***	Checklist Score	.03	.01	.26	4.57***
					Global Rating	.04	.01	.49	8.52***
5.1	Prostatic Hypertrophy								
5.2	Falling	.20	.19	18.23***	Checklist Score	.02	.01	.22	2.83**
					Global Rating	.03	.01	.35	4.50***
6	Necropsy	.04	.03	8.41**	Checklist Score	.04	.01	.19	2.90**

Legend: B unstandardised regression coefficient. SE Standard error. β standardised regression coefficient. ***p < 0.001. **p < 0.01

In four out of six OSCE stations, both the clinical skill rating and the communication skill rating positively predict the entrustment rating, with β values ranging between 0.22–0.35 and 0.21–0.52, respectively. The only station where the clinical skill rating has no significant impact on the entrustment rating is in the herniated disc station. Additionally, in four out of six stations, the communication skill rating has a greater effect on the entrustment rating than the clinical skill rating does.

### Assessors’ evaluation of the entrustment scale rating

A total of 48 assessors participated in the evaluation (response rate 89%). They had an average of 6 years (SD = 5) of experience in supervising medical students or residents.

Table 4 provides an overview of how the OSCE assessors evaluate the addition of the entrustment scale. As an indicator of feasibility, the majority indicate that its application is not time consuming. Regarding its educational value, the majority of the assessors consider the entrustment scale to be useful as a tool for evaluating students’ skills and providing individual feedback. Most of them also agree on the statement that the addition of an entrustment rating scale would be a meaningful addition to the standard OSCE assessment. The assessors remained undecided regarding the question of whether the general impression of students’ performance can be summarised with an entrustment scale (approximately one third each claimed agreement, neutrality or disagreement).

**Table 4:** Descriptive statistics of the usability questionnaire

Item	Percentage agree or fully agree	Mean	SD
The application of the entrustment rating scale is not time consuming.	77.1%	4.0	1.1
The entrustment rating scale …			
• is useful for the evaluation of the clinical and cognitive competence of the students.	70.8%	3.8	1.1
• enables feedback on individual performance.	62.5%	3.6	.9
• is in general a useful addition to the OSCE assessment form.	64.6%	3.7	1.3
• can summarize the general impression of students’ performance.	35.4%	3.1	1.1

## Discussion

This study explores the introduction of an entrustment rating scale into an existing OSCE administered before the final clerkship year in an undergraduate medical programme. Overall, we feel that the findings broaden our understanding of this matter on an empirical basis and provide directions for future, more specific studies on whether and, if so, how entrustment scales should be employed in OSCEs. In the following, we discuss the results for the three main questions raised in light of the current literature and propose topics to be addressed in subsequent studies.

Regarding question one, we found that entrustment ratings show a greater distribution across the whole scale compared to the standard OSCE ratings on clinical or communication skills, which were located mostly in the upper parts of the scales. This finding is in line with the use of an entrustment rating scale in the assessment of workplace performance [25]. However, it is not clear why raters apply the OSCE and entrustment rating differently. It has also previously been proposed that global ratings capture something different than OSCE checklists do, allowing them to better determine trainees’ level of proficiency [17]. Rekman and colleagues [26] suggest that entrustment scales are better construct-aligned scales because they reflect the expertise and priorities of clinical educators. Both the formation and the interpretation of entrustment ratings may thus be more meaningful for clinical assessors. Future qualitative studies may provide more insights into why assessor rate students differently on standard OSCE than on entrustment scales.

In further exploration of the distribution, we applied a pass threshold to the individual OSCE station results. While the non-pass number was low for the standard OSCE ratings, a substantially higher number of trainees would not have passed based on the entrustment ratings. This finding suggests that entrustment scales may identify struggling learners better, and if this is in fact the case, future studies should show this also for their actual workplace performance. Nevertheless, we feel that this is important information on trainees’ readiness for workplace participation, as determined by the OSCE assessment. On the one hand, this information can provide trainees with feedback to stimulate their future learning and skills training [27]. On the other hand, it may support clinical supervisors by indicating that certain trainees require a closer level of supervision at the beginning. Whether such evaluations should be best based on non-passing of a single or multiple OSCE stations should be addressed in future studies. With such an approach, we might be able to shift the focus from passing an exam to preparing trainees for the workplace [6].

Regarding question 2, the regression analyses indicated that in most but not all OSCE stations, the ratings of clinical and communication skills accounted for a reasonably high proportion of the variance in entrustment ratings’ variance, with the highest R^2^ score being approximately 50%. This finding is underpinned by previous research showing that the ability of a trainee to perform a clinical skill is one important factor in the entrustment decision-making of clinical supervisors. However, other factors also play a role, such as individual attributes of the trainee or supervising physician, the trainee-supervisor relationship, the task itself and the circumstances [13, 14]. Interestingly, across many stations, the communication skill rating had a greater effect on the entrustment scale rating than the clinical skill rating did. Our scale for communication skill ratings included items on structure, empathy, and non-verbal and verbal communication [20]. These items could have provided clues about trustworthiness dimensions, such as the trainee’s integrity or humility. To our knowledge, no study has estimated the effect size of the factors in a simulated setting as we have done. Future studies may undertake similar regression analyses on trainees’ task performance and entrustment ratings in a real life, clinical workplace.

Another interesting finding in this light concerns the two OSCE stations where the entrustment ratings did not relate statistically to ratings of clinical and communication skills. We have no explanation for this phenomenon; it should certainly be the object of future research. It might depend on the structure of these OSCEs, the specific performance expectations or whether the task is classified as a realistic workplace activity. In any case, this finding raises the question of whether entrustment ratings scales can be automatically introduced in any exiting OSCE station. A regression analysis, as performed in this study, may be used to provide evidence for the validity of the entrustment scale rating at a certain OSCE station.

Regarding question three, assessors provided mainly positive evaluations regarding the introduction of an entrustment scale in OSCEs. The majority of assessors agree that the entrustment scale could be a useful addition to the OSCE evaluation form, but they also showed some variability in their evaluations. These doubts should not be ignored, and efforts should be undertaken to understand them in more detail.

Overall, this study provides empirical insights on the addition of entrustment ratings scales into existing OSCEs and offers sufficient support for future research on this matter. In our institution, we plan to further explore the addition of entrustment ratings in OSCEs. In addition to including an assessor training on entrustment ratings, we plan to research the use of non-pass entrustment rating results as formative student feedback that indicates their potential insufficient readiness for the final clerkship year. We acknowledge that entrustment ratings do involve some subjectivity on the part of the assessor. This should not be perceived as problematic but should instead be taken advantage of, as it yields valuable information [28, 29]. It is advised to gather multiple entrustment ratings across various situations in simulated settings and the workplace from various assessors to gain a picture of trainees’ professional development and to decide on their readiness for practice [6, 7].

This study has limitations. It represents a single-centre study, which means that the findings might not be generalisable to other contexts. Our assessors did not undergo rater calibration training on utilising the entrustment scale. Assessors who were more familiar with the concept of EPAs and entrustment decisions might have applied the scale differently [28, 30]. We could not calculate any inter-rater variance. The study was situated in a regular OSCE context, in which students in each OSCE station are observed by just one assessor. Furthermore, this study did not explore what medical students think about the introduction of entrustment scales in the OSCE assessment.

## Conclusions

The study presented here explored the introduction of an entrustment rating scale into the assessment of an existing pre-final clerkship year OSCE. We found that assessors’ ratings on standard OSCE scales were different from their ratings on entrustment scales. The entrustment ratings were influenced by trainees’ clinical and communication skill performance, but other factors were also involved. This study generated empirical evidence for further research on this matter. Non-pass entrustment ratings may serve as formative feedback for students on insufficient readiness for practice.

## Data Availability

The dataset used and analysed during this study is available from the corresponding author on request where warranted.

## Abbreviations

CBME: Competency-based medical education
EPAs: Entrustable professional activities
OSCE: Objective structured clinical exams
